# Pulmonary Histoplasmosis

**DOI:** 10.4269/ajtmh.22-0541

**Published:** 2023-01-09

**Authors:** Juan Cataño, Jessica Porras

**Affiliations:** ^1^ Infectious Diseases Section, Internal Medicine Department, University of Antioquia School of Medicine, Medellín, Colombia;; ^2^ Infectious Diseases Section, CES Clinic, Medellín, Colombia

**Figure 1.**
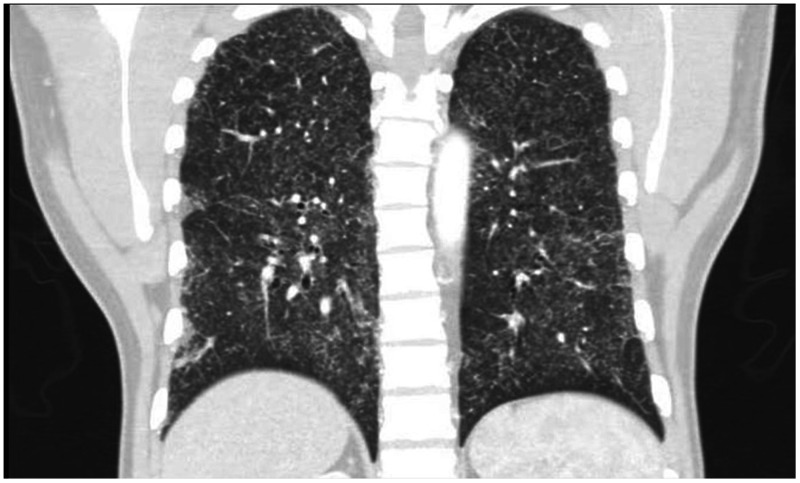
Coronal view. Contrasted chest computed tomography scan showing multiple centrilobular micronodules, with a budding tree pattern and ground glass.

**Figure 2.**
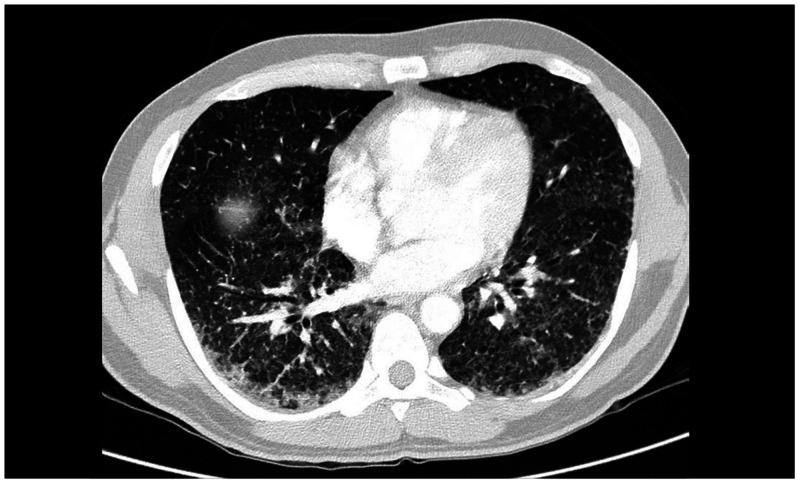
Axial view. Contrasted chest computed tomography scan showing multiple centrilobular micronodules, with a budding tree pattern and ground glass.

**Figure 3.**
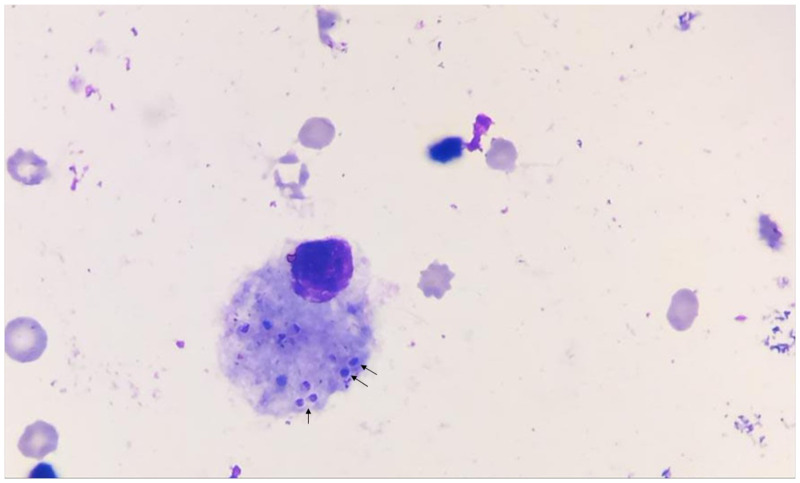
Histopathology of bronchoalveolar lavage, showing multiple macrophage cells containing intracytoplasmic yeast-like forms (arrows), consistent with *Histoplasma capsulatum.*

A 43-year-old injection drug user who had an HIV infection diagnosed in 2012 and was intermittently adherent to antiretroviral therapy presented to the infectious diseases outpatient clinic with a 3-month history of 10-kg weight loss, subjective fever, malaise, shortness of breath, productive yellowish cough, and progressive dyspnea, but no other significant related symptoms. On examination, he appeared chronically ill and wasted. Vital signs included a blood pressure level of 110/60 mm Hg, pulse rate of 115/minute, and a temperature of 38.8°C. There were no mouth lesions, and chest auscultation revealed only bibasal crackles. On abdominal examination, the liver was enlarged (span, 14 cm) but not tender, neither cervical nor axillary lymphadenopathies were found, and there were no skin lesions. The remainder of the physical examination was normal. Laboratory data showed a white blood cell count of 3.6 cells/mL (85% neutrophils); hemoglobin, 10.4 mg/dL; platelets, 155.000/mL; creatinine value, 0.4 mg/dL; HIV viral load, 964.168 copies/mL; and CD4 count, 23 cells/μL. Contrasted chest computed tomography was performed, showing multiple centrilobular micronodules, with a budding tree pattern and ground glass, mainly at the bases (Figures 1 and 2). With these images, a clinical diagnosis of tuberculosis was suspected. A bronchoalveolar lavage was performed, showing multiple macrophage cells containing 2-µm-diameter intracytoplasmic yeast-like forms (arrows), consistent with *Histoplasma capsulatum* (Figure 3), which was confirmed by specific polymerase chain reaction and culture. The patient was started on amphotericin B deoxycholate over 14 days, showing rapid improvement of symptoms. He was then sent home to continue oral itraconazole, but he never came back for follow-up.

Histoplasmosis is one of the most frequent causes of fungal respiratory infection in endemic regions; it has a broad spectrum of clinical manifestations and can present in several forms. It is highly endemic in regions of Central and South America, as well as being reported in parts of Asia and Africa.^1^ The risk of histoplasmosis is greatest in patients with severely impaired cellular immunity, especially those with HIV and CD4^+^ counts of < 150 cells/μL.^2^ A direct correlation exists between the CD4^+^ T-cell count and the capacity of macrophages to bind yeast cells. CD4^+^ cells are very important in controlling primary infection. Infected macrophages induce granuloma formation. However, macrophages from HIV-infected individuals do not mount an effective immune response, and reactivation of latent organisms is considered by some to be the common mode of infection in immunocompromised patients.^3^ Pulmonary histoplasmosis is a common manifestation of *Histoplasma* infection, with features similar to those of pulmonary tuberculosis; if it remains undiagnosed or untreated, it can also cause significant morbidity and mortality. Amphotericin B is the drug of choice for moderate to severe and disseminated presentations, whereas itraconazole is appropriate for mild disease and as step-down therapy.^4^
